# A comprehensive retrospective cohort study of the journey of B-cell lymphoma in Taiwan

**DOI:** 10.1038/s41598-021-89316-y

**Published:** 2021-05-12

**Authors:** Sung-Nan Pei, Ming-Chung Wang, Ming-Chun Ma, Ching-Yuan Kuo, Chun-Kai Liao, Hong Qiu, Lee Anne Rothwell, Yanfang Liu

**Affiliations:** 1 Division of Hematology-Oncology, Department of Internal Medicine, Kaohsiung Chang Gung Memorial Hospital, Kaohsiung, Taiwan; 2 Global Epidemiology, Janssen Research & Development, 1125 Trenton-Harbourton Road, Titusville, NJ 08560 USA; 3 Janssen Medical Affairs Asia Pacific, 66, Waterloo Road, North Ryde, NSW 2113 Australia; 4 Global Epidemiology, Janssen Research & Development, 2 Science Park Drive, Singapore, 118222 Singapore; 5 Present Address: Department of Hematology Oncology, E-Da Cancer Hospital, Kaohsiung, Taiwan; 6 Present Address: College of Medicine, I-Shou University, Kaohsiung, Taiwan

**Keywords:** Oncology, Risk factors

## Abstract

Complete disease journey and risk factors for poor outcomes are needed to facilitate effectiveness evaluations of new therapies and clinical decision-making in B-cell Non-Hodgkin lymphoma (B-NHL), particularly in Asia where such data are lacking. This retrospective cohort study used electronic medical records from a regional medical centre in southern Taiwan to follow-up 441 patients newly diagnosed with common B-NHL subtypes: Diffuse Large B-cell Lymphoma (DLBCL), Follicular Lymphoma (FL), Chronic Lymphocytic Leukaemia/Small Lymphocytic Lymphoma (CLL/SLL), Marginal Zone Lymphoma (MZL), Mantle Cell Lymphoma (MCL), and Waldenström macroglobulinemia (WM), between 01-Jan-2008 and 31-Dec-2013, until 31-Dec-2017. Treatment pathways were modelled using a Markov approach. Stage III/IV disease at diagnosis was frequent for patients with DLBCL, FL, MCL and WM. Hepatitis B surface antigen/hepatitis C virus seropositivity was 18.6%/12.3%. Clinical responses to 1st-line treatment were observed in 76.0% (DLBCL), 87.3% (FL), 86.0% (MZL), 60.0% (MCL), and 42.9% (WM) of patients. For DLBCL, disease control was achieved by ~ 50% after 1st-line, ~ 24% after 2nd-line, ~ 17% after 3rd-line. Patients with Stage III/IV DLBCL or age > 65 years at diagnosis had lower rates of active treatment, poorer disease control and higher mortality than patients with early stage disease or age ≤ 65 years. Disease flare < 6 months after 1st-line treatment was significantly associated with mortality. Despite good clinical response rates for some sub-types, survival remains poor. New treatments are needed to improve the outcome of B-NHL.

## Introduction

B-cell Non-Hodgkin lymphomas (B-NHL) are a mixed group of lymphoid malignancies that differ markedly in their clinical presentation, progression and prognosis^1^. The addition of rituximab to the treatment repertoire for B-NHL has significantly improved event-free survival (EFS) and/or overall survival (OS) across multiple subtypes^2^, and other novel agents such as ibrutinib, have also been authorized to treat B-NHL or are in development^3^. Many countries in the Asia–Pacific region do not yet have access to immune therapies other than rituximab and may be unable to apply recommended international treatment guidelines because of limitations in drug access or reimbursement policies. Only limited data describing the epidemiology, treatment and outcomes of B-NHL are available for the Asia–Pacific. Contemporary real-world data about treatment pathways and outcomes of current standards of care will be needed to understand the potential impacts of new therapies when they are introduced. Such data can inform clinical evaluations and aid in developing treatment guidelines.

We analyzed the clinical characteristics, treatment, clinical response rates and survival outcomes among patients newly diagnosed with one of the common B-NHL subtypes; Diffuse Large B-cell lymphoma (DLBCL), Follicular lymphoma (FL), Chronic Lymphocytic Leukaemia and Small Lymphocytic Lymphoma (CLL/SLL), Marginal Zone Lymphoma (MZL), Mantle Cell Lymphoma (MCL), and Waldenström macroglobulinemia (WM). We constructed disease progression models (DPMs) to visualize the treatment journey in patients with common B-NHL subtypes.

## Methods

### Study design and population

This retrospective hospital-based cohort study used electronic medical records (EMR) from the Kaohsiung Chang Gung Memorial Hospital (CGMH-KS). CGMH-KS is a 3000-bed tertiary teaching hospital in Southern Taiwan that provides daily services to over 6900 outpatients and 370 emergency patients. The hospital database holds complete inpatient, outpatient and emergency room records for each patient, including demographic and diagnostic data, laboratory and imaging reports, treatments and discharge summaries.

The study objective was to provide information about current treatment patterns of B-NHL in Taiwan, clinical outcomes of therapy and risk factors for progression. Deaths were captured from the National Death Registry which was linked to the hospital database.

The study included all patients who were ≥ 18 years of age with a record of a new diagnosis of B-NHL from January 01, 2008 until December 31, 2013 (ICD-O codes 9680/3, 9823/3, 9690/3, 9673/3, 9699/3, 9761/3). Patients with a record of any other prior malignancy were excluded. Patients were followed up until death, loss to follow-up, or study end (31-Dec-2017), whichever occurred first.

### Baseline characteristics and outcome variables

Information about demographic characteristics, disease features, treatments for B-NHL, results of laboratory testing, clinical outcomes, and date and cause of death were extracted from the EMR database. Anti-HCV antibodies were measured on serum samples using commercial kits (Abbott ARCHITECT anti-HCV, Abbott GmbH&Co., Germany).

The documented response for each patient was based on the assessment of the treating physician. Lymphoma staging workup in our institute includes whole body CT or PET-CT, plus bone marrow trephine biopsy. The interim response is typically evaluated with CT scan after 3–4 cycles of chemotherapy; and final response evaluated after the full course of 1st-line therapy with whole body CT or PET-CT. Those with initial bone marrow involvement would undergo bone marrow biopsy to confirm the clinical response. Clinical outcomes were defined using internationally recognized response criteria^4,5^. A complete response (CR) was defined as disappearance of all evidence of disease; partial response (PR) as ≥ 50% decrease in the sum of the diameters of measurable lesions; and stable disease (SD) as failure to attain CR/PR or progressive disease (PD)^4^. Clinical response definitions for WM incorporated measurement of serum monoclonal IgM as well as resolution of extramedullary disease. A CR required the absence of monoclonal IgM and normal serum IgM levels, and PR a ≥ 50% reduction in serum IgM^5^.

For the assessment of EFS, events were defined as disease progression during 1st-line treatment, disease progression after PR, relapse after CR, initiation of a new anti-cancer treatment, death from any cause (lymphoma progression or complication or unknown) or development of a second primary malignancy^6^. If a patient had more than one event, only the earliest event was counted. EFS was calculated from the first date of therapy to the first event. OS was calculated for all deaths across the study period.

### Statistical analysis

OS and EFS were estimated using Kaplan–Meier methods. EFS was not evaluated in patients with CLL/SLL because response criteria are different for CLL and SLL. This category includes a mixed patient population with disease across the spectrum of leukemia-dominant to lymphoma-dominant manifestations. Since treatment for each patient is individually selected according to the type of disease, it was not feasible to determine the EFS in this mixed group.

DPMs were constructed using a Markov model of the probabilities of different treatment states and the rates of transitions among them^7^. We calculated the probability of different disease states, including transition from one line of treatment to the next, and death. Results were displayed using Sankey diagrams with horizontal and vertical nodes that displayed treatment transition from 1st-line to 3rd-line treatments in patients with B-NHL. The DPMs illustrate the percentage of patients with each B-NHL subtype who moved from 1st-line to 2nd-line through to the end of 3rd-line treatment and includes those who died during their disease journey. Arrows that return to the line of therapy indicate patients who remained on that line of therapy until study end. Arrows to ‘Death’ indicate the percentage of patients who died at each treatment line. Mean and median show the gap in months between commencement of one line of therapy and the next. The length, width and area of each component in the figure is directly proportional to the numbers displayed. SAS 9.4 was used to generate the variables and Microsoft Visio was used to draw the graphs.

Potential risk factors for events were evaluated for DLBCL, the most prevalent subtype, using a Cox regression model. The risk of death was evaluated in patients who experienced disease flare after 1st-line treatment. Patients who experienced early disease flare, defined as relapse within 6 months after the last dose of 1st-line chemotherapy were observed from the flare date^8^. To control for immortal time bias, the time period from the last prescription for 1st-line treatment to the early flare date was calculated. For patients who experienced a disease flare at least 6 months after 1st-line treatment, the time 0 was set and matched with that of patients who experienced an early flare. Risk associated with early disease flare was adjusted in a Cox proportional hazard model by age, sex, disease stage and anti-HCV antibody status. In view of the limited sample size of the study population, these covariates were identified from the literature (Supplement). Statistical analyses were performed using SAS 9.4.

### Ethics approval

The study protocol was approved by the Chang Gung Medical Foundation Institutional Review Board with an exemption granted from the need for patient consent. Data were de-identified and patient privacy was maintained. The study was conducted in accordance with relevant guidelines and regulations.

## Results

### Baseline characteristics

There were 441 patients newly diagnosed with B-NHL at CGMH-KS between 2008 and 2013 (Tables 1, S1, and S2). Stage III/IV disease was present at diagnosis in 51.9% of patients with DLBCL, 90.0% with MCL, 68.7% with FL, 16.4% with MZL, and 100% with WM. Spleen/extra-nodal site involvement was common. Elevated lactate dehydrogenase was present at diagnosis in 64.3% of patients with DLBCL, 42.6% with CLL/SLL, 50.0% with MCL, 44.8% with FL, 24.6% with MZL, and 13.3% with WM. There were 18.6% (78 among 419 patients tested) of patients who were hepatitis B surface antigen (HBsAg) positive and 12.3% (51 among 413 tested) who were anti-hepatitis C virus (HCV) antibody positive. HCV positivity was highest (15.8%) in patients with DLBCL.

**Table 1:** Baseline demographic characteristics of patients with B-NHL in Kaohsiung Chang Gung Memorial Hospital (2008–2013).

	DLBCL	CLL/SLL	MCL	FL	MZL	WM
N	241 (100)	47 (100)	10 (100)	67 (100)	61 (100)	15 (100)
**Age (years)**						
Mean (SD)	60.6 (17.1)	63.9 (12.1)	63.7 (10.1)	59.5 (13.7)	59.3 (14.3)	66.8 (13.7)
Median (min–max)	63.6 (17.4–91.2)	64.4 (40.6–85.9)	61.5 (50.4–82.6)	57.9 (29–95)	60 (23.8–87)	68.5 (41.3–88.9)
**Age (years) group, n (%)**						
≤ 65	127 (52.7)	24 (51.1)	7 (70.0)	48 (71.6)	40 (65.6)	7 (46.7)
> 65	114 (47.3)	23 (48.9)	3 (30.0)	19 (28.4)	21 (34.4)	8 (53.3)
**Sex, n (%)**						
Male	119 (49.4)	30 (63.8)	6 (60)	29 (43.3)	28 (45.9)	9 (60)
Female	122 (50.6)	17 (36.2)	4 (40)	38 (56.7)	33 (54.1)	6 (40)
**Clinical stage, n (%)**						
I	54 (22.4)	NA	0 (0)	12 (17.9)	36 (59)	0 (0)
II	59 (24.5)	NA	1 (10)	9 (13.4)	10 (16.4)	0 (0)
III	51 (21.2)	NA	2 (20)	14 (20.9)	1 (1.6)	0 (0)
IV	74 (30.7)	NA	7 (70)	32 (47.8)	9 (14.8)	15 (100)
Missing	3 (1.2)	NA	0 (0)	0 (0)	5 (8.2)	0 (0)
**Extranodal site, n (%)**						
0	96 (39.8)	NA	2 (20)	28 (41.8)	6 (9.8)	0 (0)
1	78 (32.4)	NA	1 (10)	24 (35.8)	51 (83.6)	7 (46.7)
> 1	64 (26.6)	NA	7 (70)	15 (22.4)	4 (6.6)	7 (46.7)
Missing	3 (1.2)	NA	0 (0)	0 (0)	0 (0)	1 (6.7)
**Spleen involvement, n (%)**						
Yes	53 (22)	NA	5 (50)	14 (20.9)	6 (9.8)	5 (33.3)
No	186 (77.2)	NA	5 (50)	53 (79.1)	55 (90.2)	10 (67.7)
Missing	2 (0.8)	NA	0 (0)	0 (0)	0 (0)	0 (0)
**Liver involvement, n (%)**						
Yes	31 (12.9)	NA	2 (20)	7 (10.4)	1 (1.6)	1 (6.7)
No	208 (86.3)	NA	8 (80)	60 (89.6)	60 (98.4)	14 (93.3)
Missing	2 (0.8)	NA	0 (0)	0 (0)	0 (0)	0 (0)
**B symptoms, n (%)**						
Yes	64 (26.6)	4	5 (50)	9 (13.2)	4 (6.6)	7 (46.7)
No	169 (70.1)	–	5 (50)	56 (83.8)	56 (91.8)	8 (53.3)
Missing	8 (3.3)	–	0 (0)	2 (3)	1 (1.6)	0 (0)
**ANC (/μL)**						
Mean (SD)	5664.8 (3568.1)	5678.2 (3469.8)	4399.1 (2512.06)	4357.4 (2282.5)	6694.9 (18,006.7)	4605.4 (2959.5)
Median (Min–Max)	4711.2 (195–23,750)	4804.2 (896–21,831)	4354 (627.0–9125.6)	3784.1 (570–12,607.1)	3729.5 (1018–133,270.8)	3888 (1650–11,495.4)
Missing n (%)	0 (0)	0 (0)	0 (0)	3 (4.5)	9 (14.8)	0 (0)
**ALC (/μL)**						
Mean (SD)	1480.7 (1161.9)	35,351.7 (59,385.8)	5233.5 (8833.6)	2479.9 (3454.1)	1956.7 (648.1)	1612.6 (910.2)
Median (Min–Max)	1315.8 (56–13,050)	17,025 (982.8–280,023.4)	1660.7 (348–29,473.5)	1676.4 (330–21,252)	1990.4 (260–3577.3)	1421.4 (400–3763)
Missing n (%)	0 (0)	0 (0)	0 (0)	4 (6.0)	3 (6.1)	0 (0)
**Creatinine (mg/dL)**						
Mean (SD)	0.94 (0.71)	1.1 (0.7)	0.9 (0.29)	0.95 (0.61)	0.9 (0.5)	1.13 (0.53)
Median (Min–Max)	0.8 (0.28–8.04)	0.9 (0.4–4.7)	0.84 (0.4–1.47)	0.84 (0.26–4.11)	0.8 (0.4–3.7)	1.18 (0.29–1.97)
Missing n (%)	0 (0)	6 (12.8)	0 (0)	1 (1.5)	1 (1.6)	0 (0)
**ALT (U/L)**						
Mean (SD)	38.8 (59.4)	32.2 (43)	20.6 (9.61)	23.56 (17.9)	26 (15.1)	31.7 (43.5)
Median (Min–Max)	23 (6–541)	17 (8–233)	18 (9–37)	18 (4.3–121)	22 (9–98)	22 (5–184)
Missing n (%)	5 (2.1)	8 (17.0)	0 (0)	2 (3.0)	4 (6.6)	0 (0)
**LDH (normal vs > normal), n (%)**						
Normal	77 (32)	20 (42.6)	5 (50)	35 (52.2)	35 (57.4)	11 (73.3)
Elevation	155 (64.3)	20 (42.6)	5 (50)	30 (44.8)	15 (24.6)	2 (13.3)
Missing	9 (3.7)	7 (14.9)	0 (0)	2 (3.0)	11 (18.0)	2 (13.3)
**HBsAg positive, n (%)**						
Yes	47 (19.5)	8 (17)	3 (30)	8 (11.9)	9 (14.8)	3 (20.0)
No	188 (78)	28 (59.6)	6 (60)	59 (88.1)	48 (78.7)	12 (80.0)
Missing	6 (2.5)	11 (23.4)	1 (10)	0 (0)	4 (6.6)	0 (0)
**Anti**–**HCV positive, n (%)**						
Yes	38 (15.8)	3 (6.4)	0 (0)	5 (7.5)	3 (4.9)	2 (13.3)
No	195 (80.9)	31 (66.0)	10 (100)	60 (89.6)	54 (88.5)	12 (80.0)
Missing	8 (3.3)	13 (27.7)	0 (0)	2 (3.0)	4 (6.6)	1 (6.7)
**ECOG PS, n (%)**						
0–1	179 (74.3)	42 (89.4)	8 (80)	60 (89.6)	55 (90.2)	6 (40.0)
2–4	57 (23.7)	4 (8.5)	2 (20)	4 (6)	3 (4.9)	7 (46.7)
Missing	5 (2.1)	1 (2.1)	0 (0)	3 (4.5)	3 (4.9)	2 (13.3)

*ALC* absolute lymphocyte count, *ALT* alanine transferase, *ANC* absolute neutrophil count, *CLL/SLL* small lymphocytic lymphoma and chronic lymphocytic, *DLBCL* diffuse large B-cell lymphoma, *ECOG PS* Eastern Cooperative Group performance score, *FL* follicular lymphoma, *HBsAg* hepatitis B surface antigen, *HCV* hepatitis C virus, *LDH* lactate dehydrogenase, *SD* standard deviation, *MCL* mantle cell lymphoma, *MZL* marginal zone lymphoma, *WM* Waldenström’s macroglobulinemia or lymphoplasmacytic lymphoma.

### Treatment and clinical response rates

**Figure 1:**
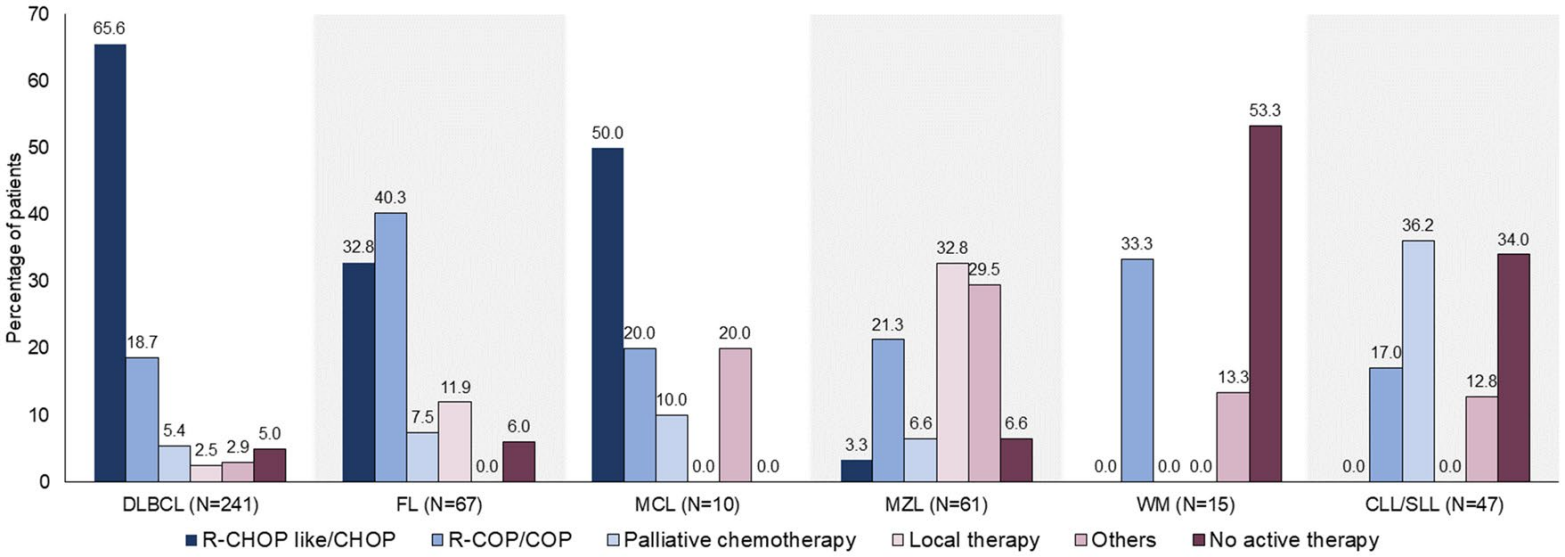
Distribution of the most common 1st-line treatments used in 6 B-NHL subtypes. Note that 1.66% patients received CHOP without R and 2.49% received COP without R. Drug abbreviations are provided in Table S4.

There were 44 patients who did not receive active treatment; 12 patients with DLBCL, one with FL and 2 with WM, all judged too weak to receive treatment; and 16 with CLL/SLL, 3 with FL, 4 with MZL and 6 with WM in whom a watch-and-wait-policy was indicated (Table S3). Treatment distribution across subtypes is shown in Fig. 1 and Table S4.

**Figure 2:**
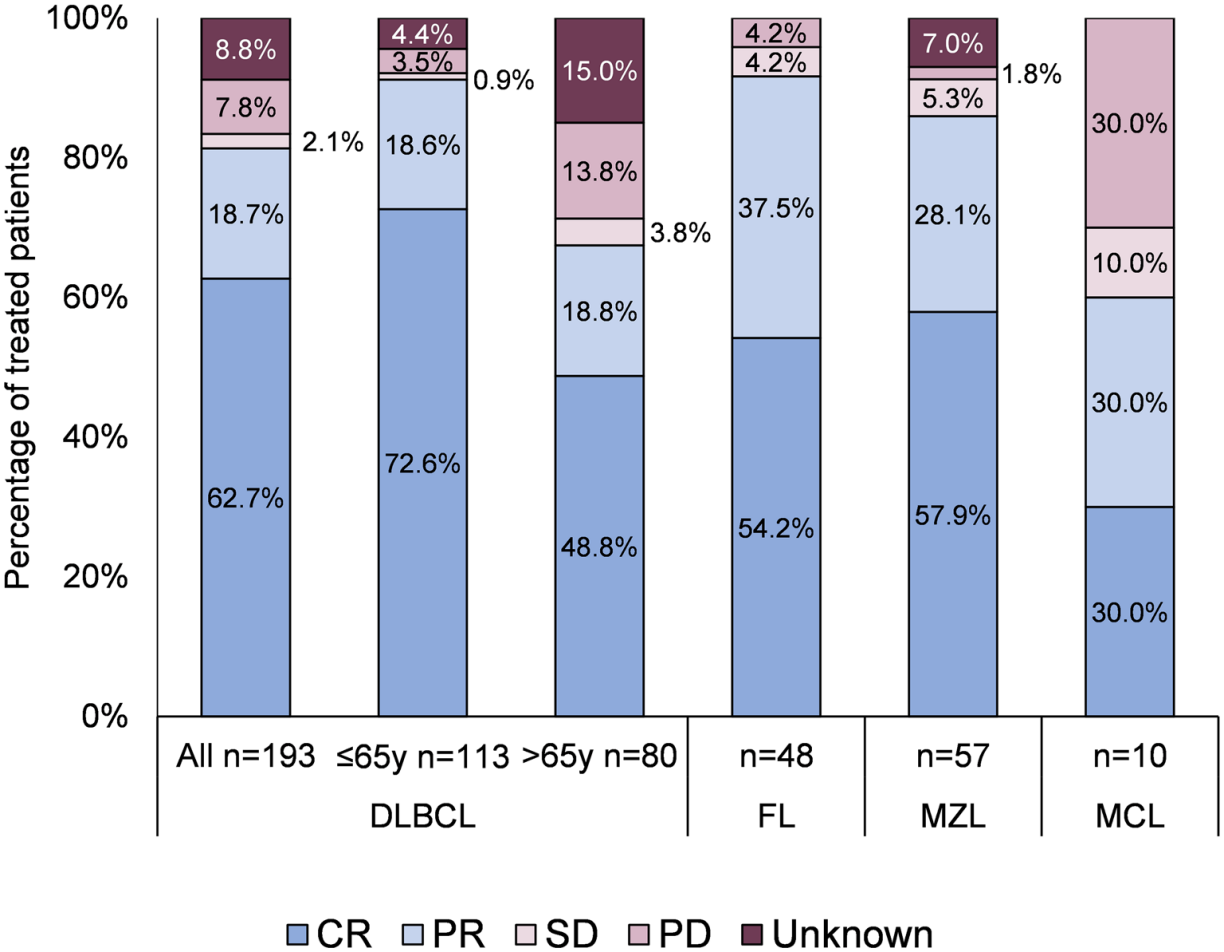
Response to 1st-line treatment among B-NHL subtypes. CR, complete response; DLBCL, diffuse large B-cell lymphoma; FL, follicular lymphoma; MCL, mantle cell lymphoma; MZL, marginal zone lymphoma; PD, progressive disease; PR, partial response; SD, stable disease. *1st-line treatment limited to R-CHOP-like/CHOP or R-COP in patients with DLBCL; R-CHOP-like/ or R-COP/COP in patients with FL; all administered treatments for other subtypes. Clinical response criteria differ for WM or CLL/SLL and these subtypes are not included in this graph. Data are tabulated and drug abbreviations explained in Table S4.

Among 193 (80.8%) patients with DLBCL who received 1st-line treatment with R-CHOP-like (R-CHOP, R-CVOP where V is for etoposide, and R-CEOP where E is for epirubicin)/CHOP or R-COP/COP (Fig. 1), 81.3% achieved a CR/PR (Fig. 2, Table S5). In 48 patients with FL who received 1st-line treatment with R-CHOP-like or R-COP/COP regimens (Fig. 1), 91.7% achieved CR/PR (Fig. 2).

Among 61 patients with MZL, 49 had extra-nodal mucosa-associated lymphoid tissue (MALToma), 6 nodal MZL, and 6 splenic MZL. The locations of MALToma were predominantly stomach/duodenum (n = 26), ocular adnexa (n = 9), and others. In patients with MZL, 1st-line therapy was an R-CHOP-like/R-COP regimen in 24.6%, local therapy in 32.8% and *Helicobacter pylori* eradication therapy in 29.1%. CR/PR after 1st-line was achieved by 86% of patients.

Treatment regimens in 10 patients with MCL were heterogeneous, ranging from very aggressive (hyperCVAD) in one patient, R-CHOP-like/CHOP in 5, bendamustine and rituximab in one, COP in 2, to palliative oral chemotherapy in one. Overall, the clinical response rate was 60%.

In 7 patients with WM who received treatment, 5 received R-COP/COP and 3 (42.9%) achieved clinical response (all PR).

Among 47 patients with CLL/SLL, 10 had an initial B-lymphocyte count ≤ 5000/μL and were diagnosed as SLL. Two adopted a watch and wait policy and 4 were treated with COP. For 37 patients with CLL, almost 40% did not require immediate therapy. Among patients who received treatment, chlorambucil and prednisolone was the most frequently used regimen (15/23) (Fig. 1).

### Treatment events after 1st-line therapy and survival

**Figure 3:**
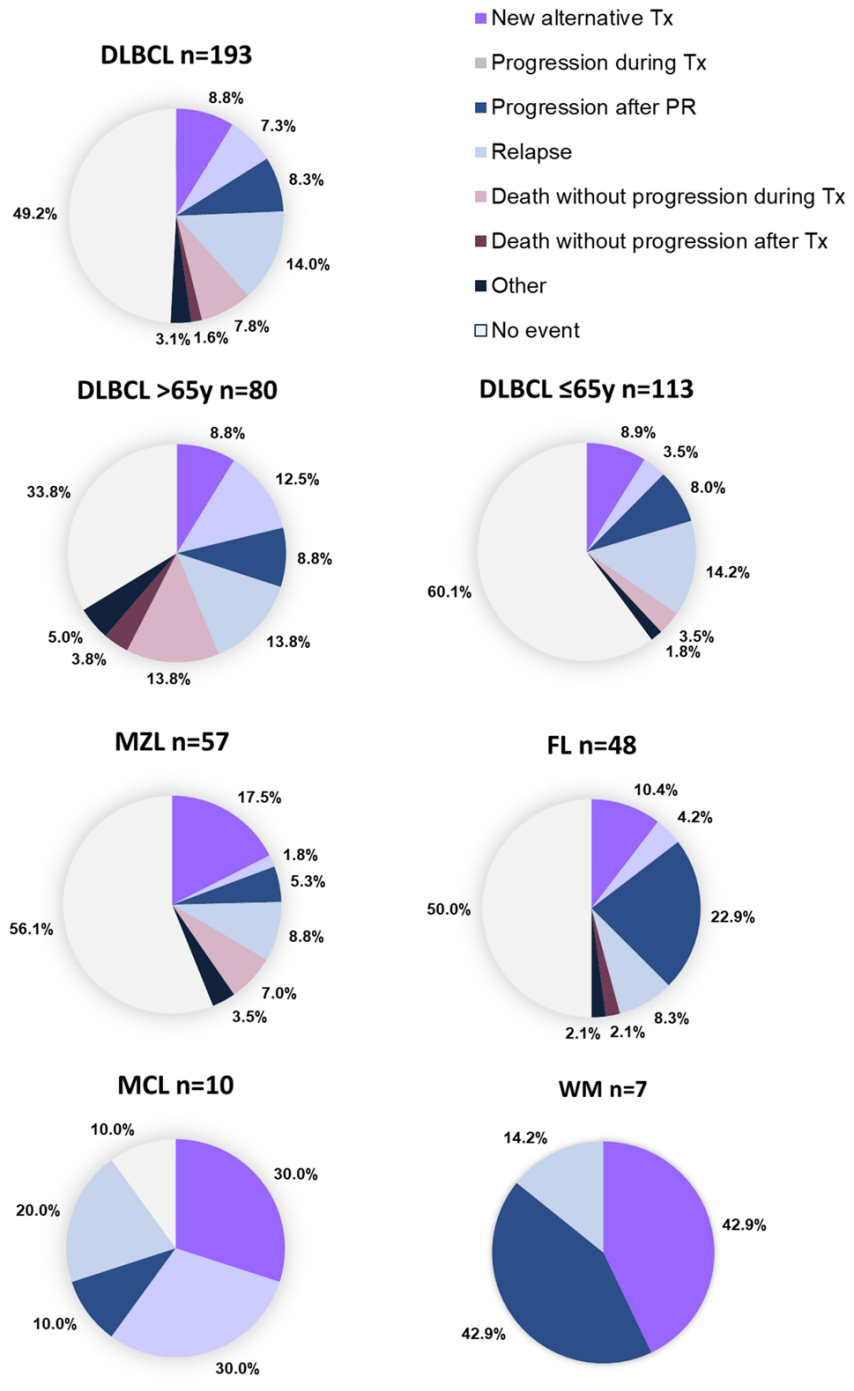
Treatment events after 1st-line treatment using standard regimens in patients with B-NHL (N = 366). DLBCL, diffuse large B-cell lymphoma; FL, follicular lymphoma; MCL, mantle cell lymphoma; MZL, marginal zone lymphoma; WM, Waldenström’s macroglobulinemia or lymphoplasmacytic lymphoma; Tx, treatment; PR, partial response. *1st-line = R-CHOP like/CHOP or R-COP/COP in patients with DLBCL; R-CHOP-like or R-COP/COP in patients with FL; all administered treatments for other subtypes.

**Figure 4:**
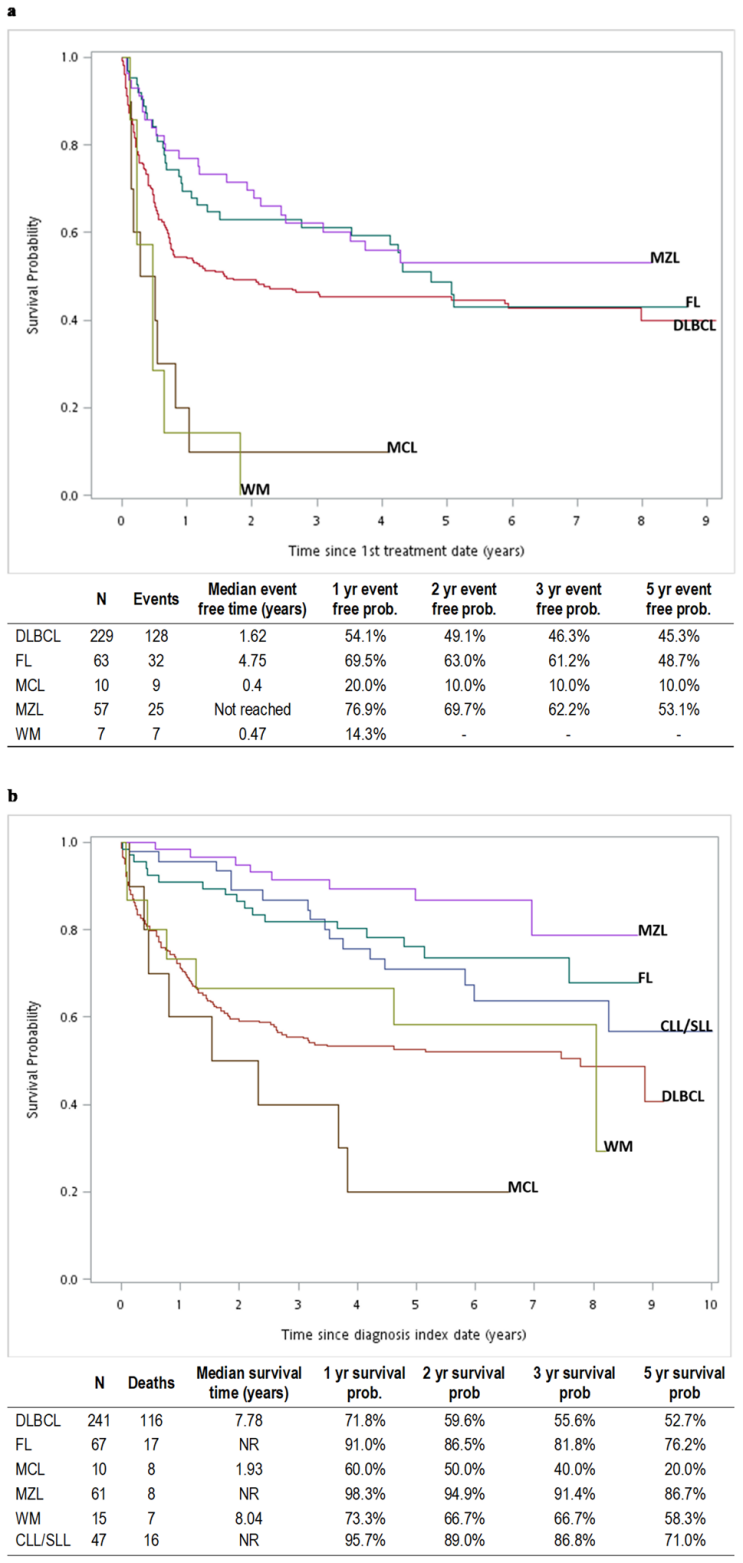
Survival in patients with 6 B-NHL subtypes (**a**) event-free survival after the date of first treatment* (**b**) overall survival in all patients after the diagnosis index date (death from any cause). CLL/SLL, small lymphocytic lymphoma and chronic lymphocytic; DLBCL, diffuse large B-cell lymphoma; FL, follicular lymphoma; MCL, mantle cell lymphoma; MZL, marginal zone lymphoma; WM, Waldenström’s macroglobulinemia or lymphoplasmacytic lymphoma.

Among patients with DLBCL, 49.2% experienced no event during/after 1st-line therapy, 7.3% progressed during 1st-line treatment, 8.3% progressed after PR, 14.0% relapsed and 9.4% died without progression during or after treatment (Fig. 3). There were 8.8% who received a new alternative regimen during 1st-line therapy, suggesting an unsatisfactory clinical response or intolerable adverse effects. Median EFS was 1.62 years (95% CI 0.76–5.94) and 5-year EFS was 43.5% (Fig. 4). Over a median follow-up period of 3.17 years, 5-year OS was 52.7% and median OS was 7.78 years.

In patients with FL, 50.0% experienced no further event during the study period, 4.2% progressed during 1st-line treatment, 10.4% received a new unplanned regimen, 22.9% progressed after PR, 8.3% relapsed and only 1 patient (2.1%) died without progression (Fig. 3). Median EFS was 4.75 years (95% CI 2.77-not estimable) and 5-year EFS was 48.7%. 5-year OS was 76.2% and median OS was not reached over the median follow-up period of 4.91 years (Fig. 4).

Among patients with MZL, 56.1% experienced no further event during the study period, 17.5% underwent a new (unplanned) anticancer treatment, and 8.8% relapsed. Few patients progressed during treatment (1.8%) or after PR (5.3%), and 10.5% died without progression during or after treatment (Fig. 3). Median EFS was not reached and 5-year EFS was 53.1%. Five-year OS was 86.7% and median OS was not reached over the median follow-up period of 4.99 years (Fig. 4).

Outcomes in patients with MCL were poor: 90% (9/10 patients) experienced an event after 1st-line treatment; 3 patients underwent a new (unplanned) anticancer treatment, 3 progressed during treatment, 1 progressed after PR and 2 died without progression during treatment. Median EFS was 0.4 years (95% CI 0.14–0.82) and 5-year EFS was 10.0%. The median study follow-up period was 1.93 years. 5-year OS was 20.0% and median OS was 1.93 years (Fig. 4).

Among 7 patients with WM, 3 underwent a new (unplanned) anticancer treatment, 3 progressed after PR and one relapsed. Median EFS was 0.47 years (95% CI 0.22–0.64) and 5-year EFS was 0%. The median study follow-up period was 4.62 years. 5-year OS was 58.3% and median OS was 8.04 years (Fig. 4).

In patients with CLL/SLL, 5-year OS was 71.0% and median OS was not reached over the median follow-up period of 5.29 years (Fig. 4).

### Risk factors impacting clinical outcome in DLBCL

Age (> 65/ ≤ 65 years), disease stage at diagnosis (I/II, III/IV) and anti-HCV antibody status were risk factors for events and death in patients with DLBCL (Table S6). Older patients (> 65 years) had more events after 1st-line treatment than younger patients (66.2% vs 39.1%) (Fig. 3). Rates of new unplanned treatments, progression after PR and relapse were similar in younger and older patients, but older patients > 65 years had higher rates of progression during treatment (12.5% vs 3.5%), and death without progression during or after treatment (27.6% vs 3.5%). For patients attaining PR/CR after R-CHOP/R-COP, age was not a risk factor for relapse (Table S7).

**Figure 5:**
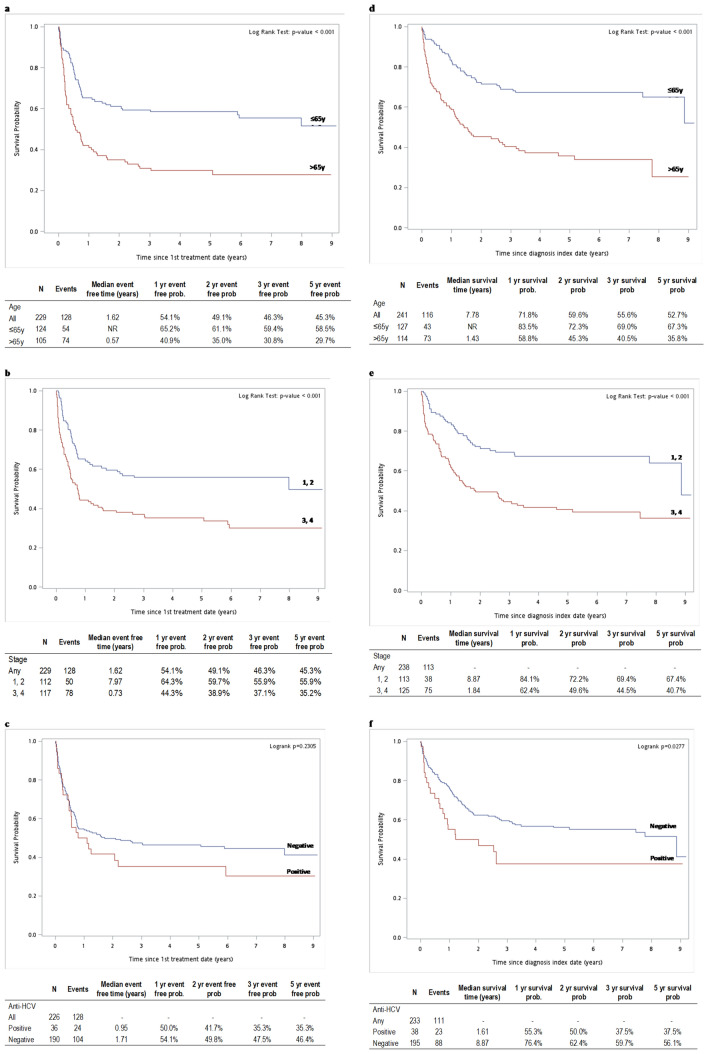
Survival in patients with DLBCL: median event-free time after the date of first treatment by (**a**) age, (**b**) stage at diagnosis, (**c**) anti-HCV antibody status. Overall survival after the diagnosis index date (death from any cause) by (**d**) age, (**e**) stage at diagnosis, (**f**) anti-HCV antibody status.

Median EFS was not reached in patients ≤ 65 years of age at diagnosis, versus 0.57 years in patients aged > 65 years and 5-year EFS was 67.3% vs 35.8%, respectively (Fig. 5a). 5-year survival was 67.3% in ≤ 65 years of age and 35.8% in > 65 year-olds, and median OS was not reached vs 1.43 years, respectively (Fig. 5d).

Patients with stage III/IV disease at diagnosis had lower median EFS and 5-year EFS (1.84 years, 40.7%) than patients with Stage I/II disease (8.87 years, 67.4% (Fig. 5b).Five-year survival was 40.7% in patients with Stage III/IV disease vs 67.4% in patients with Stage I/II disease, and median OS was 1.84 years vs 8.87 years, respectively (Fig. 5e).

Similarly, median EFS and 5-year EFS were lower in patients who were anti-HCV antibody positive at diagnosis (0.95 years, 35.3%) than anti-HCV antibody negative patients (1.71 years, 46.4%) (Fig. 5c). Five-year survival was 37.5% vs 56.1% and median OS was 1.61 versus 8.87 years, respectively (Fig. 5f).

Of 157 patients with DLBCL who achieved CR/PR after R-CHOP/R-COP 1st-line therapy, 47 experienced relapse. The crude HR for increased death in patients who experienced an early disease flare compared with patients in whom a disease flare occurred > 6 months was 2.742 (95% CI 1.253–5.998, *p* = 0.0116). The HR adjusted for age sex, disease stage and anti-HCV antibody status was 3.719 (95% CI 1.530–9.037, *p* = 0.0037). Clinical characteristics at the occurrence date of flare in patients by time to onset after CR/PR are provided in Table S8.

### Disease progression models

The DPMs illustrate probability of transition from diagnosis through lines of therapy until death for patients with DLBCL, FL and CLL/SLL.

Among 241 patients with DLBCL, 5% died without intervention due to old age, severe comorbidity or died before definitive pathology was available. 229 patients received 1st-line therapy and 51.8% achieved disease control (i.e., they were alive after 1st-line treatment and did not require 2nd-line therapy); 23.8% died during or after 1st-line therapy; 47 received 2nd-line therapy of whom 23.4% achieved disease control, while 40.4% died. For 17 relapsed patients still fit for 3rd-line therapy, only 3 could be salvaged by treatment. In our database, DLBCL 5-year survival was 52.7%.

**Figure 6:**
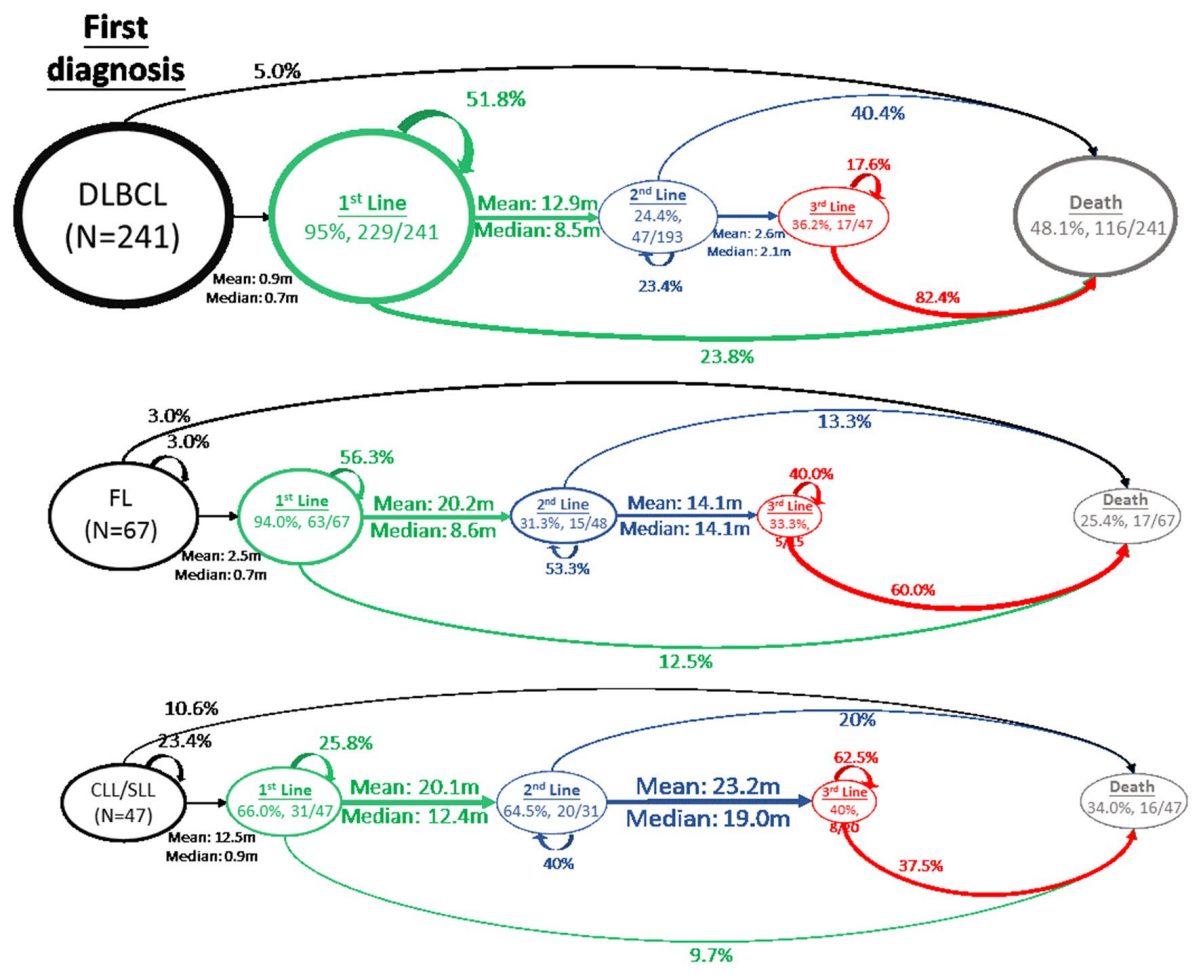
Disease progression models for DLBCL, FL and CLL. CLL/SLL, small lymphocytic lymphoma and chronic lymphocytic; DLBCL, diffuse large B-cell lymphoma; FL, follicular lymphoma. SAS 9.4 was used to calculate the probability of transition and duration of lines of treatments and Microsoft Visio was used to draw the graph.

Among patients with FL, one was too weak to treat and died and 3 had no indication for lymphoma therapy. Of these, 2 remained progression-free at last follow-up and 1 died for reasons unrelated to lymphoma. Due to the indolent nature of FL, around 50% of patients achieved disease control after each line of therapy. Amongst 63 treated patients, 31.3% progressed to 2nd-line and 33.3% of these progressed to 3rd-line (Fig. 6). 12.5% of patients died after 1st-line, 13.3% died after 2nd-line, and 60.0% died after 3rd-line. Overall, 25.4% of patients with FL died over the study period.

The indolent nature of CLL/SLL is evident in the DPM. More than one-third (n = 16) of patients diagnosed with CLL/SLL were not treated, of whom 5 died and 11 continued untreated until study end. Among treated patients, 64.5% progressed to 2nd-line and 40% progressed to 3rd-line (Fig. 6). A substantial percentage remained on 2nd and 3rd-line treatment. 9.7% of patients died after 1st-line, 20% died after 2nd-line, and 37.5% died after 3rd-line. Overall, 34.0% of patients with CLL/SLL died over the study period.

**Figure 7:**
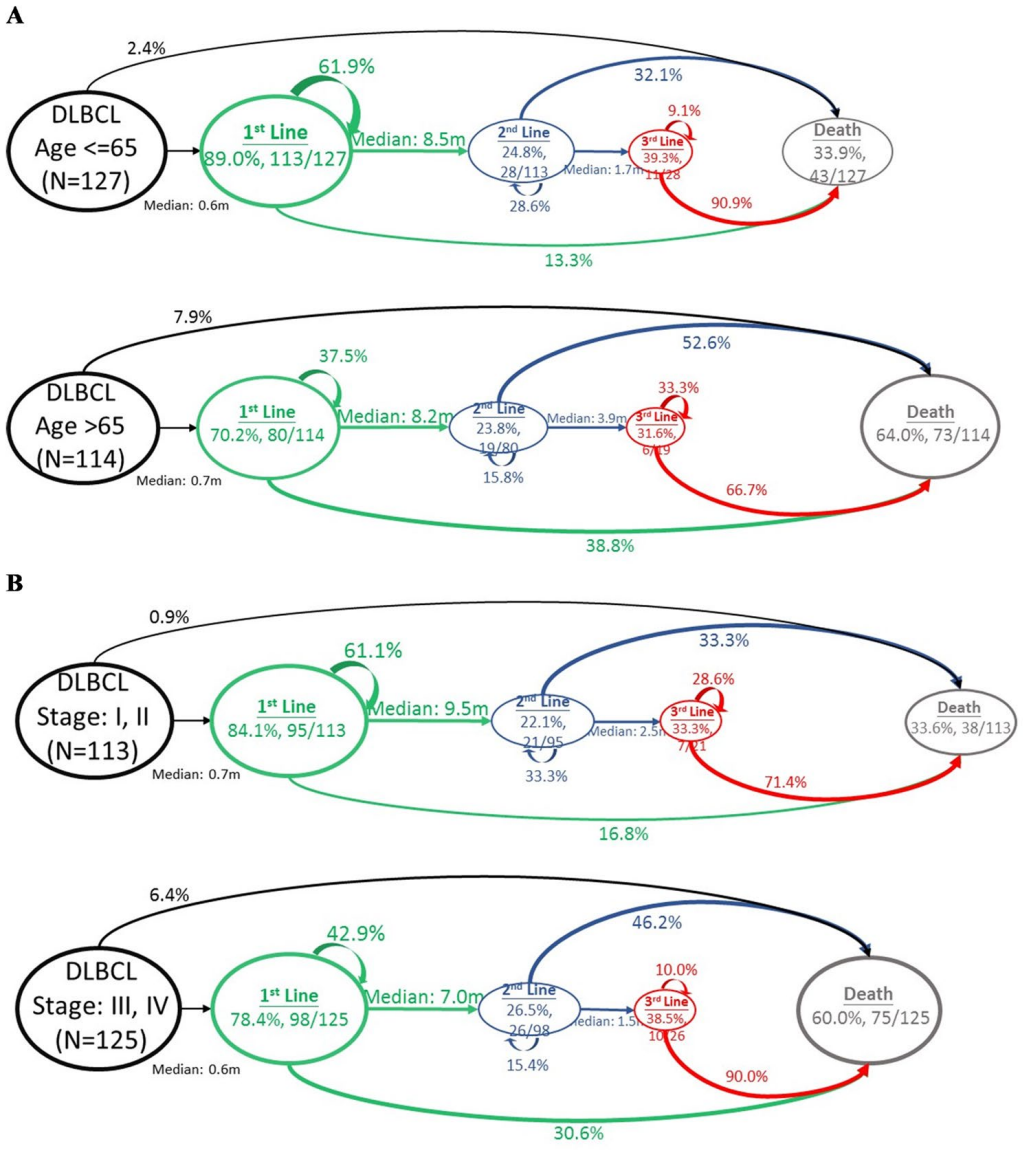
Disease progression model for DLBCL by (**A**) age and (**B**) disease stage at diagnosis. DLBCL, diffuse large B-cell lymphoma.

The disease journey was visualized in patients with DLBCL, the subtype with the largest number of patients, by age ≤ / > 65 years and stage (I/II versus III/IV) at diagnosis (Fig. 7A, B). Compared to patients aged > 65 years at diagnosis, younger patients with DLBCL had higher rates of active treatment (89.0% vs 70.2%) and had lower mortality after 1st- and 2nd-lines of therapy. Similar proportions of patients in each age-group moved to 2nd or 3rd-line. Overall, 33.9% of patients aged ≤ 65 years died during the study period versus. 64.0% of patients were > 65 years of age.

Compared to patients diagnosed with stage III/IV DLBCL, more patients with stage I/II disease received active 1st-line treatment (84.1% vs 78.4%), more achieved disease control, and fewer patients died (16.8% vs 30.6%) or moved to next line of therapy (22.1% vs 26.5%). Among patients too weak to be treated, one had Stage II and 11 had Stage III/IV disease. The initial stage was also associated with the outcome of 2nd-line therapy. For those diagnosed with early stage disease, fewer patients died after 2nd-line and fewer needed 3rd-line therapy. However, for those experiencing relapse twice, the outcome was dismal irrespective of their initial stage. Overall 33.6% of patients with Stage I/II disease died during the study period versus 60.0% of patients with Stage III/IV disease.

## Discussion

This study provides a detailed clinical picture of the characteristics, management and outcomes of a large cohort of patients with newly diagnosed B-NHL. Strengths of this study are the use of an EMR database that is rich in clinical data, containing comprehensive longitudinal information about clinical features, detailed sequential treatment regimens and treatment response throughout the entire disease journey that is rarely able to be obtained in population-based studies. Clinical outcome and survival reflect responses to real-world treatment for individual B-NHL subtypes, including patients who may not have totally adhered to treatment timelines or dose, and including those who did not receive active treatment either because of frailty, or a decision to observe (watch and wait policy). Patients in these categories are usually excluded from clinical trials. Linking the EMR to the death registry allowed us to capture all deaths regardless of where they occurred, which is frequently a limitation of hospital-based studies. The study enrolment period ended in 2013. Since then there has been virtually no change in the available treatment for B-NHL in Taiwan until very recently. Thus, our study documents the disease journey under current clinical practices and can be used as a baseline from which to evaluate new treatments.

Our study confirms regional differences in the distribution of B-NHL subtypes, with more of our B-NHL patient population having DLBCL and MZL, and fewer having FL and CLL/SLL compared to populations outside of Asia^9,10^. The percentage of patients with DLBCL who presented to our institution with extra-nodal disease, B-symptoms and advanced (Stage III–IV) diseases was similar to other reports^11^. However, compared to studies in the United States and United Kingdom, patients in Taiwan tended to be younger at the time of diagnosis, with an age-distribution similar to that observed in Hong Kong^10,12^. Younger age at B-NHL diagnosis has been associated with a lower level of industrialization for reasons that are unknown^13,14^ but which could relate to high incidences of viral hepatitis which is a known etiological factor for some lymphomas^15^.

The presence of advanced disease (Stage III/IV), age > 65 years, or anti-HCV antibody positivity at diagnosis, all negatively impacted EFS and OS in patients with DLBCL. HCV and HBV are implicated as causal agents in the development of B-cell lymphomas^15^. Consistent with this observation, 12.3% of patients with B-NHL were anti-HCV antibody positive, which is substantially higher than 4.4% seroprevalence recorded in the general population of Taiwan^16^.

The HBsAg seropositivity rate of 18.6% is higher than 13.7% seroprevalence in the general population^17^. In Taiwan, the risk of developing NHL was found to be 4.14-fold higher in persons with HBV infection, and there is evidence that the incidence of NHL in young persons has reduced since HBV vaccination introduction^18^.

Epstein-Barr virus (EBV) is a lymphotrophic virus that has been implicated in the pathogenesis of lymphoproliferative disorders including DLBCL^19^. The seroprevalence of EBV in Taiwan is high, being > 95% during adulthood^20^. However, serologic and tumor tissue testing for EBV in patients with BNHL were not routinely conducted in Taiwan during the study period (2008 to 2013) and the EBV status of patients with B-NHL could not be evaluated in our study. Since 2016, the revised World Health Organization classification groups DLBCL into EBV-positive and EBV-negative cases^21^. As EBV tissue testing becomes routine, further research may be possible to investigate the role of EBV in BNHL in Taiwan.

Relapse < 12 months after completing R-CHOP treatment in patients with DLCBL is reported to have a worse prognosis and can be challenging to manage^22^. In our adjusted analysis the risk of death in patients who experienced a disease flare after a clinical response was significantly higher (by 4.8-fold) when the flare occurred within 6 months of completing 1st-line treatment. We did not identify any demographic or disease features that differentiated patients with early flare and predicting which patients likely to experience early relapse is difficult based on clinical features alone^23^.

Using real-world data to construct DPMs allowed dynamic visualization of the treatment journey in patients with different B-NHL subtypes, highlighting the vast differences in the disease journey associated with each of these subtypes. DPMs showed how the presence of risk factors such as advanced age, disease stage at diagnosis, anti-HCV antibody positivity and early disease flare impacted the disease journey in DLBCL, including the progression between lines of therapy and mortality at each treatment stage. DPMs can be used to investigate the impact of treatment at different stages of the disease journey and could be used to identify study endpoints during the investigation of new treatments for B-NHL. The disease journeys represented here are specific to the Taiwan population which tended to be younger at diagnosis than some other countries, with high rates of HBsAg and anti-HCV antibody positivity. While possibly relevant to other parts of Asia, the results might not be applicable to other populations where other risk factors are more prevalent. Our study was limited to a single institution. Treatment data might be lost (or incomplete) if patients transferred to other hospitals, although this is unlikely given that CGMH-KS is the regional tertiary center for Southern Taiwan. Another potential limitation relates to the retrospective study design for which EMRs may not always be complete; and that we did not include other rare B-NHL subtypes or subtypes with imprecise pathological descriptions.

More than half of patients with B-NHL presenting to a tertiary hospital in Taiwan had advanced disease at diagnosis. While clinical response rates to 1st-line treatments were high in DLCBL, FL and MZL, response rates in WM and MCL were low, and survival in patients with aggressive B-NHL subtypes such as DLBCL and aggressive MCL remains poor. Variations in clinical outcome according to disease subtype, age, stage, anti-HCV antibody status and early disease flare were marked. DPMs are a tool that provide an overview of the disease journey and can be used to help manage treatment, with potentially wider application in evaluating treatment effectiveness.

## Supplementary Information

Supplementary Information 1.
