# Supplementary material for: A comprehensive retrospective cohort study of the journey of B-cell lymphoma in Taiwan
Source: Sci Rep. 2021 May 12;11:10069. doi: 10.1038/s41598-021-89316-y (PMC8115261; doi:10.1038/s41598-021-89316-y)
Supplement: Supplementary file 1 — Supplementary Information 1. [file 41598_2021_89316_MOESM1_ESM.pdf]

A Comprehensive Retrospective Cohort Study of the Journey of B-cell Lymphoma in Taiwan ~~Journey of B-cell Lymphoma: A Comprehensive Retrospective Cohort Study in Taiwan~~

Sung-Nan Pei, Ming-Chung Wang, Ming-Chun Ma, Ching-Yuan Kuo, Chun-Kai Liao, Hong Qiu, Lee Anne Rothwell, Yanfang Liu

Supplementary file

Literature consulted and used to identify covariates for inclusion in the Cox model

Alvarez, R., *et al*. What determines therapeutic choices for elderly patients with DLBCL? Clinical findings of a multicenter study in Portugal. *Clin Lymphoma Myeloma Leuk* **14,** 370-379 (2014).

Barta, S. K., *et al.* Treatment factors affecting outcomes in HIV-associated non-Hodgkin lymphomas: a pooled analysis of 1546 patients. *Blood* **122,** 3251-3262 (2013).

Besson, C., *et al.* Characteristics and outcome of diffuse large B-cell lymphoma in hepatitis C virus-positive patients in LNH 93 and LNH 98 Groupe d'Etude des Lymphomes de l'Adulte programs. *J Clin Oncol* **24**, 953-960 (2006).

Fields, P. A. and D. C. Linch. Treatment of the elderly patient with diffuse large B cell lymphoma. *Br J Haematol* **157**, 159-170 (2012).

Hamlin, P. A., *et al*. Treatment patterns and comparative effectiveness in elderly diffuse large B-cell lymphoma patients: a surveillance, epidemiology, and end results-medicare analysis. *Oncologist* **19,** 1249-1257 (2014).

Khan, A. E., *et al.* Diabetes and the risk of non-Hodgkin's lymphoma and multiple myeloma in the European Prospective Investigation into Cancer and Nutrition. *Haematologica* **93**, 842-850 (2008).

Lin, T. L., et al. The impact of age, Charlson comorbidity index, and performance status on treatment of elderly patients with diffuse large B cell lymphoma. *Ann Hematol* **91,** 1383-1391 (2012).

Morrison, V. A., *et al*. Diffuse large B-cell lymphoma in the elderly: impact of prognosis, comorbidities, geriatric assessment, and supportive care on clinical practice. An International Society of Geriatric Oncology (SIOG) expert position paper. *J Geriatr Oncol* **6**, 141-152 (2015).

van der Poel, M. W., *et al.* Factors that influence treatment decision-making in elderly DLBCL patients: a case vignette study. *Ann Hematol* **94**, 1373-1379 (2015).

Wang, F., *et al.* Clinical and prognostic analysis of hepatitis B virus infection in diffuse large B-cell lymphoma. *BMC Cancer* **8,** 115 (2008).

Williams, J. N., *et al.* Disease characteristics, patterns of care, and survival in very elderly patients with diffuse large B-cell lymphoma. *Cancer* **121,** 1800-1808 (2015).

Table S1. Baseline demographic characteristics of patients with B-NHL in Kaohsiung Chang Gung Memorial Hospital (2008-2013)

	DLBCL	CLL/SLL	MCL	FL	MZL	WM
N	241 (100)	47 (100)	10 (100)	67 (100)	61 (100)	15 (100)
**Age (years) group, n (%)**						
18 - 49	62 (25.7)	7 (14.9)	0 (0)	16 (23.9)	14 (23)	2 (13.3)
50 - 59	39 (16.2)	9 (19.2)	3 (30)	23 (34.3)	16 (26.2)	3 (20)
60 - 69	57 (23.7)	16 (34)	5 (50)	16 (23.9)	17 (27.9)	3 (20)
70 - 79	58 (24.1)	9 (19.2)	1 (10)	5 (7.5)	11 (18)	5 (33.3)
≥ 80	25 (10.4)	6 (12.8)	1 (10)	7 (10.5)	3 (4.9)	2 (13.3)
**Year of enrolment, n (%)**						
2008	38 (15.8)	5 (10.6)	0 (0)	8 (11.9)	7 (11.5)	0 (0)
2009	45 (18.7)	11 (23.4)	2 (20)	12 (17.9)	13 (21.3)	3 (20)
2010	33 (13.7)	10 (21.3)	0 (0)	13 (19.4)	8 (13.1)	4 (26.7)
2011	37 (15.4)	8 (17)	3 (30)	6 (9)	10 (16.4)	1 (6.7)
2012	38 (15.8)	5 (10.6)	1 (10)	16 (23.9)	9 (14.8)	3 (20)
2013	50 (20.8)	8 (17)	4 (40)	12 (17.9)	14 (23)	4 (26.7)
**Body weight (kg)**						
Mean (SD)	60.1 (11.9)	63.1 (11.7)	65.7 (10.4)	63 (11.6)	62.2 (12.5)	57.3 (13)
Median (min-max)	59 (31-93.5)	63 (39-95)	66.3 (53-85)	63 (38-92)	61.8 (36.8-99)	56 (40-82)
Missing n (%)	3 (1.2)	0 (0)	0 (0)	0 (0)	0 (0)	0 (0)
**Body height (cm)**						
Mean (SD)	161 (8.7)	162 (8.4)	162.9 (4.9)	161.4 (8.3)	161.6 (8.7)	160.8 (10.7)
Median (Min-Max)	160.6 (144-194)	163.1 (144-179.3)	163 (155-170)	161.1 (145-184.4)	161.3 (142.2-189)	161.6 (139.3-181)
Missing n (%)	2 (0.8)	1 (2.1)	0 (0)	0 (0)	0 (0)	1 (6.7)
**Body surface area (m^2)**						
Mean (SD)	1.6 (0.2)	1.7 (0.2)	1.7 (0.1)	1.7 (0.2)	1.7 (0.2)	1.6 (0.2)
Median (Min-Max)	1.6 (1.0-2.1)	1.7 (1.3-2.0)	1.7 (1.5-1.9)	1.7 (1.2-2.0)	1.7 (1.2-2.3)	1.6 (1.3-2.0)
Missing n (%)	3 (1.2)	1 (2.1)	0 (0)	0 (0)	0 (0)	1 (6.7)
**Liver cirrhosis (before lymphoma), n (%)**						
Yes	13 (5.4)	2 (4.3)	0 (0)	0 (0)	3 (4.9)	1 (6.7)
No	227 (94.2)	45 (95.7)	10 (100)	67 (100)	58 (95.1)	14 (93.3)
Missing	1 (0.4)	0 (0)	0 (0)	0 (0)	0 (0)	0 (0)
**White blood cell count (/µL)**						
Mean (SD)	7996 (4166.8)	43025.5 (63486.5)	11360 (10755.484)	9895.5 (16340.2)	21483.3 (79149.2)	6807 (3150)

	DLBCL	CLL/SLL	MCL	FL	MZL	WM
Median (Min-Max)	7000 (1300-32100)	24000 (5100-292300)	8000 (1900-40100)	6150 (1000-131300)	6300 (2100-590200)	6800 (2500-13800)
Missing n (%)	0 (0)	0 (0)	0 (0)	1 (1.5)	1 (1.6)	0 (0)
**Neutrophils (%)**						
Mean (SD)	69 (14.5)	25.1 (16.9)	47.16 (20.83)	59.83 (17.91)	70.5 (107.1)	64.5 (14.9)
Median (Min-Max)	70.5 (5-95)	21 (2.5-78.1)	51.35 (16.8-73.2)	61.6 (3-88.7)	59.7 (0.5-854.3)	62.4 (31.0-90.0)
Missing n (%)	0 (0)	0 (0)	0 (0)	2 (3)	4 (6.6)	0 (0)
**Lymphocyte (%)**						
Mean (SD)	20.6 (12)	70.6 (18.4)	33.09 (21.84)	28.23 (13.61)	34.5 (18.8)	26.2 (13.5)
Median (Min-Max)	18.7 (1-75)	74 (15.6-95.8)	26.15 (4.0-73.5)	29.1 (28126)	32.7 (35916)	29 (6.0-53.0)
Missing n (%)	0 (0)	0 (0)	0 (0)	3 (4.5)	4 (6.6)	0 (0)
**Anti-HBc positivity, n (%)**						
Positive	165 (68.5)	23 (48.9)	8 (80)	41 (61.2)	36 (59.0)	9 (60.0)
Negative	37 (15.4)	4 (8.5)	1 (10)	11 (16.4)	13 (21.3)	2 (13.3)
Missing	39 (16.2)	20 (42.6)	1 (10)	15 (22.4)	12 (19.7)	4 (26.7)

anti-HBc, antibodies to hepatitis B core antigen; CLL/SLL, small lymphocytic lymphoma and chronic lymphocytic; DLBCL, diffuse large B-cell lymphoma; FL, follicular lymphoma; SD, standard deviation; MCL, mantle cell lymphoma; MZL, marginal zone lymphoma; WM, Waldenström macroglobulinemia or lymphoplasmacytic lymphoma.

Table S2. Baseline prognostic scores in patients with DLBCL or FL at Kaohsiung Chang Gung Memorial Hospital (2008-2013)

B-NHL subtype	Index score		n	%
**DLBCL**	International Prognostic Index (IPI)			
		0	23	9.5
		1	59	24.5
		2	55	22.8
		3	49	20.3
		4	35	14.5
		5	14	5.8
		Missing	6	2.5
	Age-adjusted International Prognostic Index (aaIPI)			
		0	53	22.0
		1	62	25.7
		2	74	30.7
		3	38	15.8
		Missing	14	5.8
**FL**	FLIPI-1 (follicular lymphoma IPI)			
		0	6	9.0
		1	16	23.9
		2	17	25.4
		3	12	17.9
		4	9	13.4
		5	5	7.5
		Missing	2	3.0
	FLIPI-1 (follicular lymphoma IPI)-1			
		0-1 (Low)	22	32.8
		2 (Intermediate)	17	25.4
		>3 (High)	26	38.8
		Missing	2	3.0
	FLIPI-2 (follicular lymphoma IPI)-2			
		0	9	13.4
		1	19	28.4
		2	11	16.4
		3	4	6.0
		4	3	4.5
		5	3	4.5
		Missing	18	26.9
	FLIPI-2 risk			
		0 (Low)	9	13.4
		1-2 (Intermediate)	30	44.8
		3-5 (High)	10	14.9
		Missing	18	26.9

Table S3. Reasons why patients with B-NHL did not receive active treatment

			Did not receive treatment		
	Total	Received active treatment	Total	Too weak	Treatment not indicated
Sub-type	N	n (%)	n (%)	n (%)	n (%)
DLBCL	241	229 (95.0)	12 (5.0)	12 (5.0)	0 (0)
FL	67	63 (94.0)	4 (6.0)	1 (1.5)	3 (4.5)
MZL	61	57 (93.4)	4 (6.6)	0 (0)	4 (6.6)
CLL/SLL	47	31 (66.0)	16 (34.0)	0 (0)	16 (34.0)
WM	15	7 (46.7)	8 (53.3)	2 (13.3)	6 (40.0)
MCL	10	10 (100)	0 (0)	0 (0)	0 (0)

CLL/SLL, small lymphocytic lymphoma and chronic lymphocytic; DLBCL, diffuse large B-cell lymphoma; FL, follicular lymphoma; MCL, mantle cell lymphoma; MZL, marginal zone lymphoma; WM, Waldenström macroglobulinemia or lymphoplasmacytic lymphoma.

Table S4. First-line treatment regimens for patients with one of 6 B-NHL subtypes.

First-line treatment	DLBCL (N=241) n (%)	FL (N=67) n (%)	MCL (N=10) n (%)	MZL (N=61) n (%)	WM (N=15) n (%)	CLL/SLL (N=47) n (%)
R-CHOP/RCEOP/RCVOP	154 (63.90)	22 (32.84)	2 (20)	2 (3.28)	-	-
R-COP	39 (16.18)	26 (38.81)	-	3 (4.92)	1 (6.67)	-
R-oral CP +/- Radiotherapy or OP	10 (4.15)	2 (2.99)	1 (10)	-	-	-
Rituximab alone	5 (2.07)	-	-	-	-	-
CHOP/CEOP	4 (1.66)	-	3 (30)	-	-	-
COP	6 (2.49)	1 (1.49)	2 (20)	10 (16.39)	4 (26.67)	8 (17.02)
Oral CP	3 (1.24)	1 (1.49)	-	4 (6.56)	-	-
OP + Radiotherapy	1 (0.41)	1 (1.49)	-	4 (6.56)	-	-
Radiotherapy alone	3 (1.24)	5 (7.46)	-	2 (3.28)	-	-
OP alone	2 (0.83)	2 (2.99)	-	14 (22.95)	-	-
Others*	2 (0.83)	-	1 (10)	18 (29.51)	-	-
No active therapy/Hospice/Died before therapy	12 (4.98)	4 (5.97)	-	4 (6.56)	8 (53.33)	16 (34.04)
Bendamustine and rituximab	-	1 (1.49)	1 (10)	-	-	-
Bendamustine	-	-	-	-	1 (6.67)	3 (6.38)
Chlorambucil and prednisolone	-	2 (2.99)	-	-	1 (6.67)	18 (38.3)
FC	-	-	-	-	-	1 (2.13)
Rituximab and etoposide	-	-	-	-	-	1 (2.13)

Others: R-ESHAP; HyperCVAD, *Helicobacter pylori* eradication therapy for MZL
(R)-CHOP, (Rituximab), cyclophosphamide, doxorubicin, vincristine, prednisolone
(R)-CEOP, (Rituximab), cyclophosphamide, epirubicin, vincristine, and prednisone
(R)-CVOP, (Rituximab), cyclophosphamide, etoposide, vincristine, prednisolone
OP, operation
CP, cyclophosphamide, prednisolone
FC, fludarabine, cyclophosphamide

Table S5. Response to first line treatment for B-NHL subtypes[a]

	Total	CR		PR		SD		PD		Unknown	
Subtype	N	n	% (95% CI)	n	% (95% CI)	n	% (95% CI)	n	% (95% CI)	n	% (95% CI)
DLBCL (R-CHOP-like/R-COP)	193	121	62.7 (55.9-69.5)	36	18.7 (13.2-24.2)	4	2.1 (0.1-4.1)	15	7.8 (4.0-11.6)	17	8.8 (4.8-12.8)
≤65 years of age	113	82	72.6 (64.3-80.8)	21	18.6 (11.4-25.8)	1	0.9 (0.0-4.8)	4	3.5 (0.1-7.0)	5	4.4 (0.6-8.2)
>65 years of age	80	39	48.8 (37.8-59.7)	15	18.8 (10.2-27.3)	3	3.8 (0.0-7.9)	11	13.8 (6.2-21.3)	12	15.0 (7.2-22.8)
FL (R-CHOP-like/R-COP)	48	26	54.2 (40.1-68.3)	18	37.5 (23.8-51.2)	2	4.2 (0-9.8)	2	4.2 (0-9.8)	0	-
MZL	57	33	57.9 (45.1-70.7)	16	28.1 (16.4-39.7)	3	5.3 (0-11.1)	1	1.8 (0-5.2)	4	7.0 (0.4-13.7)
MCL	10	3	30.0 (1.6-58.4)	3	30.0 (1.6-58.4)	1	3.1 (0.8-5.3)	3	30.0 (1.6-58.4)	0	-

N, number of treated patients; n/%, number/percent of patients with the indicated clinical response; CI, confidence interval; CR, complete response; DLBCL, diffuse large B-cell lymphoma; FL, follicular lymphoma; MCL, mantle cell lymphoma; MZL, marginal zone lymphoma; PD, progressive disease; PR, partial response; SD, stable disease.

[a]Waldenström macroglobulinemia/lymphoplasmacytic lymphoma and small lymphocytic lymphoma and chronic lymphocytic are not applicable here

Table S6 Risk factors associated with events and death in patients with DLBCL (Cox regression model)

		HR (95% CI)	Event P-value	HR (95% CI)	Death p-value
Gender	Male	1.1 (0.8-1.5)	0.7267	1.2 (0.8-1.7)	0.3215
	Female	Ref	-	Ref	-
1st line treatment	R-CHOP-like	Ref	-	Ref	-
	R-COP	4 (2.6-6.0)	<0.0001	3.4 (2.1-5.5)	<0.0001
aaIPI	0	Ref	-	Ref	-
	1	1.7 (0.9-3.1)	0.0834	1.2 (0.6-2.3)	0.5803
	2	2.6 (1.5-4.5)	0.0010	2.5 (1.4-4.5)	0.0024
	3	5.7 (3.1-10.2)	<0.0001	4.7 (2.5-8.8)	<0.0001
Extranodal site	> 1	2 (1.3-3.1)	0.0009	2.8 (1.8-4.5)	<0.0001
(0:1:>1)	1	1.5 (0.98-2.2)	0.0642	1.9 (1.2-3.0)	0.0081
	0	Ref	-	Ref	-
Liver Cirrhosis	Yes	4.1 (2.3-7.5)	<0.0001	6 (3.3-10.8)	<0.0001
	No	Ref	-	Ref	-
Spleen	Yes	1.9 (1.3-2.7)	0.0007	1.3 (0.9-2.0)	0.1713
involvement	No	Ref	-	Ref	-
Liver involvement	Yes	2 (1.3-3.1)	0.0028	2.6 (1.7-4.2)	<0.0001
	No	Ref	-	Ref	-
B symptoms	Yes	2.4 (1.7-3.4)	<0.0001	2.1 (1.4-3.1)	0.0002
	No	Ref	-	Ref	-
Age group	> 60	2.1 (1.4-3.0)	<0.0001	2.9 (1.9-4.5)	<0.0001
	≤ 60	Ref	-	Ref	-
Age group	> 65	2.3 (1.6-3.2)	<0.0001	2.7 (1.8-3.9)	<0.0001
	≤ 65	Ref	-	Ref	-
Age group	> 70	2.5 (1.8-3.5)	<0.0001	2.7 (1.9-3.9)	<0.0001
	≤70	Ref	-	Ref	-
Stage	I	Ref	-	Ref	-
	II	1.6 (0.9-2.8)	0.1022	1.9 (0.98-3.7)	0.0582
	III	2.1 (1.2-3.7)	0.0073	2.3 (1.2-4.5)	0.0154
	IV	3.2 (1.9-5.4)	<0.0001	4.4 (2.4-8.8)	<0.0001
Stage	I-II	Ref	-	Ref	-
	III-IV	2.1 (1.5-2.9)	<0.0001	2.3 (1.6-3.4)	<0.0001
ANC Group	≥ 7,300	1.8 (1.2-2.6)	0.0032	1.9 (1.3-2.8)	0.0024
	< 7,300	Ref	-	Ref	-
ANC Group 2	≥ 4,711.2 (median)	1.6 (1.1-2.2)	0.0102	1.3 (0.9-1.8)	0.1957
	< 4,711.2 (median)	Ref	-	Ref	-
ALC Group	≥ 1,315.8 (median)	Ref	-	Ref	-
	< 1,315.8 (median)	2 (1.4-2.8)	<0.0001	1.8 (1.2-2.6)	0.0018
AST Group	≥ 74 (2*UL (37))	2 (1.2-3.3)	0.0050	2.7 (1.7-4.5)	<0.0001
	< 74 (2*UL (37))	Ref	-	Ref	-
ALT Group	≥ 80 (2*UL (40))	1 (0.6-1.9)	0.9055	0.9 (0.4-1.8)	0.6651
	< 80 (2*UL (40))	Ref	-	Ref	-
Creatinine	≥ 1.4	2.8 (1.6-4.8)	0.0002	4.9 (2.9-8.2)	<0.0001
	< 1.4	Ref	-	Ref	-
Calcium	≥ 10.0	1.3 (0.7-2.4)	0.3789	1.7 (0.9-3.2)	0.0773
	< 10.0	Ref	-	Ref	-
LDH	Elevation	2.1 (1.4-3.1)	0.0004	1.7 (1.1-2.6)	0.015
	Normal	Ref	-	Ref	-
HBsAg positive	Yes	0.6 (0.4-1.0)	0.0677	0.6 (0.4-1.1)	0.0771
	No	Ref	-	Ref	-
Anti-HCV positive	Yes	1.3 (0.9-2.1)	0.1799	1.7 (1.1-2.6)	0.0295

	No	Ref	-	Ref	-
Anti-HBs positive	Positive	1 (0.7-1.4)	0.9823	1 (0.6-1.4)	0.8143
	Negative	Ref	-	Ref	-
Anti-HBc positive	Positive	0.9 (0.6-1.4)	0.6237	0.9 (0.5-1.5)	0.6117
	Negative	Ref	-	Ref	-
ECOG	0-1	Ref	-	Ref	-
	2-4	3.4 (2.4-4.9)	<0.0001	3.6 (2.4-5.3)	<0.0001
NLR (ALC/ANC)	< 3.769	Ref	-	Ref	-
	≥ 3.769	2.6 (1.8-3.6)	<0.0001	2.5 (1.7-3.6)	<0.0001
Body weight	< 54 kg	1.8 (1.1-2.9)	0.0166	2 (1.2-3.5)	0.0121
(Female)	≥ 54 kg	Ref	-	Ref	-
Body weight	< 65 kg	1.4 (0.9-2.3)	0.1502	1.4 (0.8-2.2)	0.2435
(Male)	≥ 65 kg	Ref	-	Ref	-
Body height	< 155 cm	1.2 (0.8-1.9)	0.4218	1.6 (0.9-2.7)	0.1091
(Female)	≥ 155 cm	Ref	-	Ref	-
Body height	< 167.5 cm	1.2 (0.8-1.9)	0.4239	1.3 (0.8-2.1)	0.3344
(Male)	≥ 167.5 cm	Ref	-	Ref	-
BMI	< 18.5	1.8 (1.1-2.9)	0.0207	1.5 (0.9-2.7)	0.1218
	18.5-27	Ref	-	Ref	-
	> 27	1 (0.6-1.5)	0.8334	1 (0.6-1.7)	0.8898
BMI (Female)	< 18.5	1.6 (0.8-3.0)	0.1908	1.4 (0.7-3.0)	0.3516
	18.5-27	Ref	-	Ref	-
	> 27	1 (0.5-1.8)	0.9635	1 (0.5-2.0)	0.9387
BMI (Male)	< 18.5	2.2 (1.0-4.6)	0.0421	1.7 (0.7-4.1)	0.2031
	18.5-27	-	-	-	-

aaIPI, Age-adjusted International Prognostic Index; BMI, body mass index; CI, confidence interval; HR, crude hazard ratio; ECOG PS, Eastern Cooperative Oncology Group Performance Status score; NLR (ALC/ANC), neutrophil:lymphocyte ratio (absolute lymphocyte count over absolute neutrophil count); SD, standard variation; HBsAg, hepatitis B surface antigen; HCV, hepatitis C virus; LDH, lactate dehydrogenase

Table S7. Baseline demographic and clinical characteristics of 157 patients with DLBCL treated with R-CHOP/R-COP who achieved a complete or partial response, and in those that did/did not experience an early or late disease flare

		CR/PR			p-value
	DLBCL (N=157)	No flare (N=110)	Flare > 6m (N=17)	Flare < 6m (N=30)	
**Age (years)**					0.7495
Mean (SD)	55.9 (16.6)	54.6 (15.9)	60.1 (16.3)	58.3 (19.1)	
Median (min-max)	58.3 (17.4-85.7)	55.4 (17.4-85.7)	64.7 (20.9-80.8)	60.0 (18.4-83.4)	
**Age (years) group, n (%)**					0.1668
18 - 49	53 (33.8)	40 (36.4)	5 (29.4)	8 (26.7)	
50 - 59	28 (17.8)	21 (19.1)	0 (0)	7 (23.3)	
60 - 69	42 (26.8)	32 (29.1)	6 (35.3)	4 (13.3)	
70 - 79	26 (16.6)	12 (10.9)	5 (29.4)	9 (30.0)	
≥ 80	8 (5.1)	5 (4.6)	1 (5.9)	2 (6.7)	
**Sex, n (%)**					0.3299
Male	79 (50.3)	58 (52.7)	6 (35.3)	15 (50.0)	
Female	78 (49.7)	52 (47.3)	11 (44.7)	15 (50.0)	
**Year of enrolment, n (%)**					0.6172
2008	24 (15.3)	16 (14.6)	4 (23.5)	4 (13.3)	
2009	35 (22.3)	26 (23.6)	4 (23.5)	5 (16.7)	
2010	22 (14.0)	16 (14.6)	3 (17.7)	3 (10.0)	
2011	24 (15.3)	16 (14.6)	3 (17.7)	5 (16.7)	
2012	25 (15.9)	20 (18.2)	1 (5.9)	4 (13.3)	
2013	27 (17.2)	16 (14.6)	2 (11.8)	9 (30.0)	
**Body weight (kg)**					0.2917
Mean (SD)	61.5 (11.5)	62.1 (11.2)	57.4 (10.9)	61.4 (13.0)	
Median (min-max)	60.2 (31-93.5)	61.3 (31-90.2)	57.0 (40.3-82.0)	57.9 (37-93.5)	
**Body height (cm)**					0.3247
Mean (SD)	161.9 (8.5)	162.6 (8.6)	158.7 (8.4)	161.2 (8.3)	
Median (Min-Max)	162 (145-194)	163 (146-194)	157 (145.5-172.0)	160 (145-174.5)	
**Body surface area (m^2)**					0.2744
Mean (SD)	1.7 (0.2)	1.7 (0.2)	1.6 (0.2)	1.6 (0.2)	
Median (Min-Max)	1.6 (1.1-2.1)	1.7 (1.1-2.1)	1.6 (1.3-2.0)	1.6 (1.2-2.1)	
**Clinical stage, n (%)**					0.3982
I	38 (24.2)	32 (29.1)	2 (11.8)	4 (13.3)	
II	47 (29.9)	31 (28.2)	7 (41.2)	9 (30.0)	
III	35 (22.3)	22 (20.0)	6 (35.3)	7 (23.3)	
IV	37 (23.6)	25 (22.7)	2 (11.8)	10 (33.3)	
**Extranodal site, n (%)**					0.3442
0	70 (44.6)	52 (47.3)	8 (47.1)	10 (33.3)	
1	52 (33.1)	34 (30.9)	7 (41.2)	11 (36.7)	
>1	35 (22.3)	24 (21.8)	2 (11.8)	9 (30.0)	
**Liver cirrhosis (before lymphoma), n (%)**					0.5282
Yes	5 (3.2)	3 (2.0)	0 (0)	2 (6.7)	
No	152 (96.8)	107 (98.0)	17 (100)	28 (93.3)	
**Spleen involvement, n (%)**					0.1762
Yes	30 (19.1)	17 (15.4)	7 (41.2)	6 (20.0)	
No	127 (80.9)	93 (84.6)	10 (58.8)	24 (80.0)	
**Liver involvement, n (%)**					0.3958
Yes	13 (8.3)	7 (6.4)	1 (5.9)	5 (16.7)	
No	144 (81.7)	103 (93.6)	16 (94.1)	25 (83.3)	
**B symptom, n (%)**					0.1762
Yes	32 (20.4)	19 (17.6)	7 (41.2)	6 (20.0)	
No	123 (79.6)	89 (82.4)	10 (58.8)	24 (80.0)	
Missing	2 (1.0)	2 (1.4)	0 (0)	0 (0)	
**White blood cell count (/μL)**					0.3522

	DLBCL (N=157)	CR/PR No flare (N=110)	Flare > 6m (N=17)	Flare < 6m (N=30)	p-value
Mean (SD)	7723.6 (3245.9)	7845.5 (3415.6)	6923.5 (2676.6)	7730 (2905.3)	
Median (Min-Max)	7000 (1300-18900)	6850 (1300-18900)	7300 (3200-12200)	7300 (3400-14200)	
**Neutrophils (%)**					0.1010
Mean (SD)	67.3 (13.2)	66.3 (13.3)	64.9 (15.7)	72.1 (9.8)	
Median (Min-Max)	67.7 (14-93.4)	65.7 (14-92)	68 (25-91.3)	72.5 (52.8-93.4)	
**ANC (/µL)**					0.0855
Mean (SD)	5326.7 (2899.8)	5363.6 (3056.9)	4399.9 (1904.5)	5716.9 (2728.2)	
Median (Min-Max)	4519.8 (715-17010)	4513.2 (715-17010)	4117.5 (1722.6-7486.6)	4773.6 (2172.6-12056)	
**Lymphocyte (%)**					0.1308
Mean (SD)	22.3 (11.4)	23.6 (12.0)	22 (10)	17.7 (8.8)	
Median (Min-Max)	21.5 (1-75)	24.0 (1-75)	19.6 (4.7-51.0)	16.2 (2.7-36.7)	
**ALC (/µL)**					0.3626
Mean (SD)	1598.6 (1182.1)	1702.1 (1308.4)	1494.3 (889.2)	1278.2 (702.5)	
Median (Min-Max)	1408 (189-13050)	1536 (189-13050)	1151.7 (385.4-3876)	1156.7 (294.3-3435.2)	
**Creatinine (mg/dL)**					0.3977
Mean (SD)	0.8 (0.3)	0.8 (0.3)	0.8 (0.3)	0.9 (0.4)	
Median (Min-Max)	0.8 (0.3-2.2)	0.8 (0.3-1.8)	0.8 (0.4-1.3)	0.8 (0.5-2.2)	
**ALT (U/L)**					0.5257
Mean (SD)	35.5 (52.7)	38.3 (61.1)	32.3 (29.3)	27.2 (16.6)	
Median (Min-Max)	22 (6-541)	23 (6-541)	19 (7-117)	21 (10-68)	
Missing n (%)	2 (1.3)	1 (0.9)	0 (0)	1 (3.3)	
**LDH (Normal vs. Elevation), n (%)**					0.2824
Normal	57 (36.3)	46 (42.6)	6 (35.3)	5 (17.2)	
Elevation	97 (61.8)	62 (57.4)	11 (64.7)	24 (82.8)	
Missing	3 (1.9)	2 (1.8)	0 (0)	1 (3.3)	
**HBsAg positive, n (%)**					0.6918
Yes	34 (21.7)	27 (24.8)	3 (17.6)	4 (13.3)	
No	122 (77.7)	82 (75.2)	14 (82.4)	26 (86.7)	
Missing	1 (0.6)	1 (0.9)	0 (0)	0 (0)	
**Anti-HCV positivity, n (%)**					0.4350
Yes	24 (15.3)	16 (14.8)	4 (23.5)	4 (13.3)	
No	131 (83.4)	92 (85.2)	13 (76.5)	26 (86.7)	
Missing	2 (1.3)	2 (1.8)	0 (0)	0 (0)	
**Anti-HBc positivity, n (%)**					0.6085
Positive	77 (49.0)	54 (54.0)	9 (56.3)	14 (48.3)	
Negative	68 (43.3)	46 (46.0)	7 (43.7)	15 (51.7)	
Missing	12 (7.7)	10 (9.1)	1 (5.9)	1 (3.3)	
**ECOG Group, n (%)**					0.4211
0-1	137 (87.3)	99 (90.0)	12 (75.0)	26 (86.7)	
2-4	19 (12.1)	11 (10.0)	4 (25.0)	4 (13.3)	
Missing	1 (0.6)	0 (0)	1 (5.9)	0 (0)	
**NLR, n (%)**					0.1035
< 3.8	64 (40.8)	40 (36.4)	6 (35.3)	18 (60.0)	
≥3.8	93 (59.2)	70 (63.6)	11 (64.7)	12 (40.0)	

ALC, absolute lymphocyte count; ALT, alanine transferase; ANC, absolute neutrophil count; anti-HBc, antibodies to hepatitis B core antigen; anti-HBs, antibodies to hepatitis B surface antigen; DLBCL, diffuse large B-cell lymphoma; ECOG, Eastern Cooperative Group; HBsAg, hepatitis B surface antigen; HCV, hepatitis C virus; LDH, lactate dehydrogenase; NRL, neutrophil:lymphocyte ratio; SD, standard deviation

Table S8. Clinical characteristics at the occurrence date of flare in 47 patients with DLBCL treated with R-CHOP/R-COP who achieved a complete or partial response

		Flare > 6m (N=17)	Flare < 6m (N=30)	p-value
**Response, n (%)**				0.5895
	CR	11 (64.7)	17 (56.7)	
	PR	6 (35.3)	13 (43.3)	
**Age at flare, n (%)**				0.2140
	<= 65	7 (41.2)	18 (60.0)	
	> 65	10 (58.8)	12 (40.0)	
**Body weight (kg)**				0.5945
	Mean (SD)	57.9 (11.6)	59.9 (12.0)	
	Median (min-max)	55.1 (37.8-80.7)	58.5 (43.1-90.0)	
	Missing n (%)	1 (5.9)	2 (6.7)	
**Body height (cm)**				0.3474
	Mean (SD)	158.5 (9.0)	161.1 (8.6)	
	Median (Min-Max)	157 (142-176)	160.1 (144.3-174.6)	
	Missing n (%)	1 (5.9)	2 (6.7)	
**Body surface area (m^2)**				0.4114
	Mean (SD)	1.6 (0.2)	1.6 (0.2)	
	Median (Min-Max)	1.5 (1.3-2.0)	1.6 (1.3-2.0)	
	Missing n (%)	1 (5.9)	2 (6.7)	
**Clinical stage, n (%)**				0.6777
	I, II	7 (50.0)	9 (42.9)	
	III, IV	7 (50.0)	12 (57.1)	
	Missing	3 (17.5)	9 (30.0)	
**IPI, n (%)**				0.5452
	0	2 (15.4)	2 (10.5)	
	1	5 (38.5)	2 (10.5)	
	2	2 (15.4)	5 (26.3)	
	3	2 (15.4)	6 (31.6)	
	4	1 (7.7)	2 (10.5)	
	5	1 (7.7)	2 (10.5)	
	Missing	4 (23.5)	11 (36.7)	
**Extranodal site, n (%)**				0.3977
	0	12 (85.7)	17 (73.9)	
	> 1	2 (14.3)	6 (26.1)	
	Missing	3 (17.5)	7 (23.3)	
**White blood cell count (/μL)**				0.2895
	Mean (SD)	5631.3 (1940)	6600 (4174.1)	
	Median (Min-Max)	5600 (2700-9300)	5300 (2400-23000)	
	Missing n (%)	1 (5.9)	0 (0)	
**Neutrophils (%)**				0.2090
	Mean (SD)	60.6 (15.2)	66.2 (13.6)	
	Median (Min-Max)	58.9 (39.5-92.6)	67.6 (40-90.3)	
	Missing n (%)	1 (5.9)	0 (0)	
**Lymphocyte (%)**				0.5071
	Mean (SD)	28.4 (12.8)	24.6 (20.3)	
	Median (Min-Max)	27.9 (4.7-48.9)	19.9 (5.9-115.8)	
	Missing n (%)	1 (5.9)	0 (0)	
**Monocyte (%)**				0.1107
	Mean (SD)	7.4 (2.5)	9.2 (5.1)	
	Median (Min-Max)	7.5 (2.6-11)	7.6 (3-25)	
	Missing n (%)	1 (5.9)	0 (0)	
**AST (U/L)**				0.1789
	Mean (SD)	34.4 (14.2)	46.4 (39.5)	
	Median (Min-Max)	27 (25-75)	31 (12-176)	
	Missing n (%)	2 (11.8)	5 (16.7)	

**ALT (U/L)**				0.4229
	Mean (SD)	33.2 (22.3)	40.6 (38.4)	
	Median (Min-Max)	29 (13-93)	28 (1-172)	
	Missing n (%)	2 (11.8)	1 (3.3)	
**Creatinine (mg/dL)**				0.9476
	Mean (SD)	0.8 (0.5)	0.8 (0.4)	
	Median (Min-Max)	0.6 (0.5-2.7)	0.8 (0.4-2.6)	
	Missing n (%)	1 (5.9)	0 (0)	
**Calcium (mg/dL)**				0.1647
	Mean (SD)	9.0 (0.4)	9.4 (0.9)	
	Median (Min-Max)	9.0 (8.2-9.7)	9.2 (8.6-12.2)	
	Missing n (%)	6 (35.3)	16 (53.3)	
**LDH (Normal vs. Elevation), n (%)**				0.0343
	Normal	7 (46.7)	4 (14.8)	
	Elevation	8 (53.3)	23 (85.2)	
	Missing	2 (11.8)	3 (10.0)	
**ECOG PS, n (%)**				0.7349
	0-1	12 (75.0)	18 (66.7)	
	2-4	4 (25.0)	9 (33.3)	
	Missing	1 (5.9)	3 (10.0)	

ALT, alanine transferase; AST, aspartate transaminase; CR, complete response; ECOG, Eastern Cooperative Group; IPI, International Prognostic Index; PR, partial response; SD, standard deviation
